# Effect of Intra-Orifice Depth on Sealing Ability of Four Materials in the Orifices of Root-Filled Teeth: An Ex-Vivo Study

**DOI:** 10.1155/2012/318108

**Published:** 2012-05-22

**Authors:** Motaz Ahmad Ghulman, Madiha Gomaa

**Affiliations:** ^1^Department of Restorative Dentistry, Faculty of Dentistry, King Abdulaziz University, Jeddah 21589, Saudi Arabia; ^2^Endodontic Division, Department of Restorative Dentistry, Faculty of Dentistry, King Abdulaziz University, Jeddah 21589, Saudi Arabia

## Abstract

*Aim*. To investigate the effect of orifice cavity depth on the sealing ability of Fusio, Fuji II, Fuji IX, and MTA“G”. *Materials and Methods*. Ninety-two canals in extracted mandibular premolars were prepared, obturated, and randomly grouped into 4 groups. Each group was subgrouped for a 2 mm and 3 mm orifice cavity depth (*n* = 10). The remaining roots were divided to serve as positive and negative controls (*n* = 6). Cavities of the 4 experimental groups were filled with the respective materials and subjected to methylene blue dye leakage. Linear leakage was measured in mm using a stereomicroscope. *Statistical Analysis*. Kruskall-Wallis test was used at *P* < 0.05, and *t*-test was done to compare 2 mm and 3 mm. *Results*. All tested materials leaked to various degrees. Significantly higher leakage score was found for Fuji IX, Fusio, Fuji II, and MTA “G” in a descending order, when the materials were placed at 3 mm depths. A significant difference was found in the leakage score between the 2 mm and 3 mm depths in all tested materials with the 3 mm depth showing a greater leakage score in all tested materials. Exception was in MTA “G” at 2 mm and 3 mm depths (0.551 mm ± 0.004 mm and 0.308 mm ± 0.08 mm, resp.). *Conclusion*. The null hypothesis should be partially rejected. Fusio and MTA “G” were affected by orifice cavity depth with regard to their sealing ability. MTA “G” had the least leakage when placed at 2 or 3 mm depths, and Fusio is the next when placed at 2 mm depth. Two millimeters orifice cavity depth is suitable for most adhesive orifice barrier materials.

## 1. Introduction

A major cause of developed or persistent apical periodontitis is coronal bacterial microleakage [1, 2]. As the intracanal obturating material—cores as well as sealers—are not leak proof, leakage is assumed to occur at the sealer-canal wall interface or the gutta-percha-sealer interface once oral fluid has reached a canal orifice [3, 4]. In addition to well-instrumented and three-dimensionally obturated root canal spaces, bacteria must be prevented from reaching the root canal system through a coronal leakage. Although endodontic cases are frequently referred for specialty care, it is actually the restorative dentist who is responsible for completion of the canal space obturation procedure [5]. Indeed preservation and protection of the canals system from leakage in the lapse of time from referral to the definitive restoration placement by the restorative dentist is mandatory. As a protection of the root canal filling from leakage prior to the subsequent restorative procedure, many temporary restorative materials were initially suggested as an interim restoration. Of these Cavit, SuperEBA, and IRM cement were frequently used [6–8]. However, these materials had the drawback that they should be placed in 3.5 mm thick layer which is not practical for most teeth. Again, the sealing capacity of most of them was found to be insufficient [9–13].

Orifice barriers technique was introduced on the basis that the use of a material to seal the orifice, in addition to the restoration, can moderate and prevent bacterial leakage if that restoration was missing or became unfunctional [14–16]. This relatively recent technique is based on replacing the gutta percha and sealer at the canal orifice(s) with a barrier material that is required to be leak proof.

In this respect, many materials were investigated and compared for their effective sealing ability at the canal orifices using different methodologies [17–22]. Of these materials amalgam, Geristore (compomer), Fuji-plus [17], MTA [17–19, 23], Tetric flow, glass ionomer cement, resin-modified glass ionomer cement [19, 20], and Cavit G [20] were all examined.

Generally, none of the previously investigated materials was capable of complete or prolonged abolishing of leakage with varying degrees. On the other hand, the depth to which these materials are inserted which reflects the orifice barrier thickness was scarcely studied [24]. It appears that this issue either was left for personal preference or is judged by the material to be used or the leakage assessment methodology.

The aim of the present study was to test the sealing ability of 4 orifice bonding materials—namely, fusio, Gray MTA (GMTA), Fuji II, and fuji IX—when placed at two different orifice cavity depths in terms of its possible effect on the sealing ability. The null hypothesis to be tested is that all experimented materials placed in the specified cavity depths leak to same extent.

## 2. Materials and Methods

### 2.1. Specimen Preparation

Ninety-two recently extracted, human mandibular premolars were used in the study. Teeth were extracted for orthodontic purposes. Inclusion criteria were that selected teeth has completely developed root apices and a single canal (type I) as verified by radiographic examination. Teeth were cleaned free from calculus and submerged in sodium hypochlorite for four hours to remove soft tissue attachment. They were then washed thoroughly under running water and kept preserved in saline ready for use in the study.

Teeth were decoronated using diamond discs under copious irrigation. Standard lengths were adjusted for all teeth roots to be 13 mm.

### 2.2. Endodontic Procedure

In a preparation for biomechanical instrumentation, working length was measured by introduction of a K-file size number 10 until it appeared flushed to the apex. This measurement was then adjusted at one millimeter shorter than the measured length. Glide path was confirmed using a size number 15 K-file to the apical constriction, and canal orifices were uniformly enlarged with Gates Glidden drills to a size number 4 (diameter of 1.1 mm) and a depth of 3 mm. Canals were then prepared using the Revo S NiTi system according to the manufacturer directions to an apical size of number 25 and taper of 6%. A new pack of instruments was used every 6-canal preparation.

5.25% solution of sodium hypochlorite was regularly used during biomechanical preparation to affect cleaning of the root canal system. Prepared canals were then flushed with a 2 mL of 17% EDTA solution followed by a final rinse with 2 mL of 5.25% solution of sodium hypochlorite to remove the smear layer. Root canal specimens were then dried with paper points and obturated using warm lateral compaction with gutta-percha and AH26 sealer using Endotec II tip.

### 2.3. Teeth Specimens Grouping and Orifice Cavity Depth Preparation

At this stage, teeth specimens were randomly grouped into four groups of 20 teeth each (*n* = 20) for the four tested orifice barrier materials. The remaining 12 teeth specimens were subdivided into 2 control groups (*n* = 6) to serve as positive and negative controls. Each group was then subdivided into two subgroups of ten teeth each according to the level of searing of gutta-percha (labeled as 2 mm or 3 mm).

Searing of the excess gutta-percha as well as vertical compaction at the canal's orifices was made to a 2 mm or 3 mm standard depths using a suitable size pluggers. This left a 2 mm or 3 mm empty canal orifice as verified by a graduated periodontal probe. This space was then scrubbed and cleaned from excess sealer using cotton pellets and alcohol. Prepared orifice cavities were flushed with a 1 mL of 17% EDTA solution followed by a final rinse with 1 mL of saline and gently air dried. Afterward obturated teeth specimens were preserved in 100% humidity in a humidor for 48 hrs to allow for complete sealer setting.

### 2.4. Restorative Procedures

Experimental Groups 1–4 were allocated for orifice barrier filling using Fusio self-adhesive flowable composite (Fusio Liquid Dentin, Pentron Clinical Technologies, LLC), Gray ProRoot MTA (Dentsply Tulsa Dental, Tulsa, OK), Fuji II (GC Corporation, Tokyo, Japan), and Fuji IX (GC Corporation, America). All restorations were placed by the author.

Each of the experimental orifice barrier material was packed to the orifice level and finished by following the respective manufacturer's directions. For the first group 1 mm increment of Fusio was syringed and agitated with the needle tip for 20 sec and light cured for 10 sec using a visible light activator (Bluephase. Ivoclar/Vivadent, Schaan, Liechtenstein). Additional material was then syringed in 1 mm or 2 mm, increment (in subgroups 2 mm and 3 mm, resp.). This was followed by light curing for 10 sec according to the manufacturer's directions. For the second group, Gray MTA was spatulated according to the manufacturer's directions, packed in increments in the assigned cavities, respectively, and excess water was blotted out to allow for a dense pack. Finally a piece of moistened cotton pellet was placed on top of filling barrier to help in accelerating the setting process. As for Group 3, Fuji II-according to the manufacturer recommendations—GC Dentin Conditioner was applied to the dentin orifice cavities for 20 seconds for cleaning of the walls. Cavities were then rinsed thoroughly with water and gently dried. Desiccation was avoided as recommended. Powder was divided into two equal parts using a plastic spatula. The first portion was incorporated into the liquid, mixed together for about 10 seconds. Then the second part was added and mixed for 10–15 seconds. Mixed material was then loaded in the C-R Syringe (Centrix Inc.), dispensed onto the assigned cavities of each subgroup, and cured for 20 seconds with a visible light curing device.

The fourth group, Fuji IX capsule, was tapped on a flat surface to fluff the powder; capsule was activated by depressing the button on the bottom before placement high-speed amalgamator where it was triturated for 10 seconds. Capsule was placed in the applier and the material was immediately delivered to the prepared orifice cavities of the assigned subgroups according to the manufacturer's directions.

The fifth group was subdivided into two subgroups of six roots each (*n* = 6) to possess negative and positive controls. In the positive control group, orifice cavities were prepared and left without intra-orifice barrier. In the negative control group, canals were obturated with gutta-percha to the orifice level.

Each tooth specimen was placed into a coded tube and preserved in 100% humidity in a humidor at 37°C for 48 hrs to allow for complete experimental materials setting.

### 2.5. Assessment Procedure

For each specimen, root apex was blocked by sticky wax. All experimental teeth specimens received three layers of nail polish from the level of the cementoenamel junction to the root apex except for an area of 1 mm around the orifice barrier. Positive controls were not coated with nail polish. Teeth specimens of the negative control group were completely coated with nail polish, including the canal orifice.

Samples were submerged in 2% methylene blue dye solution and centrifuged at 30 g for 5 minutes. They were then rinsed under running tap water for 5 minutes. Nail polish was gently removed from the root surfaces using scalpels. Samples were subsequently mounted in self curing acrylic resin using cubical wax molds. After curing, mounted root specimens were longitudinally sectioned using diamond discs under copious water spray. This resulted in two sections for each specimen.

### 2.6. Stereomicroscopic Evaluation of Dye Penetration

Root sections were observed using a stereomicroscope (Olympus) with a camera attached (Sharper Image Digital 130x USB microscope camera (San Francisco, CA, USA)). Images were transferred to the computer using computer software (Digital viewer) and saved as TEF format. Images were then analyzed using the Leica Application Suite U3.1.0 after covering the area of interest with a yellow color. Leica S8 APO Microscope and the digital camera were used to transfer the photo to the monitor. Depth of longitudinal dye penetration in mm was then measured mesial and distal to intra-orifice barrier material from the cavosurface margin inward on both specimen sections. The highest reading was recorded as the dye penetration depth. Measurements for all specimens were done blindly by one calibrated rater.

### 2.7. Statistical Analysis

Data were tabulated and subjected to statistical analysis using Kruskall-Wallis test at a confidence level of 95% (*P* < 0.05). The *t*-test for independent samples was done for each material to compare between 2 mm and 3 mm.

## 3. Results

A detailed descriptive statistics for the results of dye penetration are presented in Table 1 for the four materials tested at the two cavity depths. Positive control teeth showed complete full intra-orifice cavity depth leakage while specimens of the negative control did not show leakage. A general trend towards a higher leakage score was found when the materials were placed at 3 mm depths for Fuji IX, Fusio, Fuji II, and MTA “G” in a descending order (Table 1). This difference was highly significant (*P* < 0.001).

**Figure 1:**
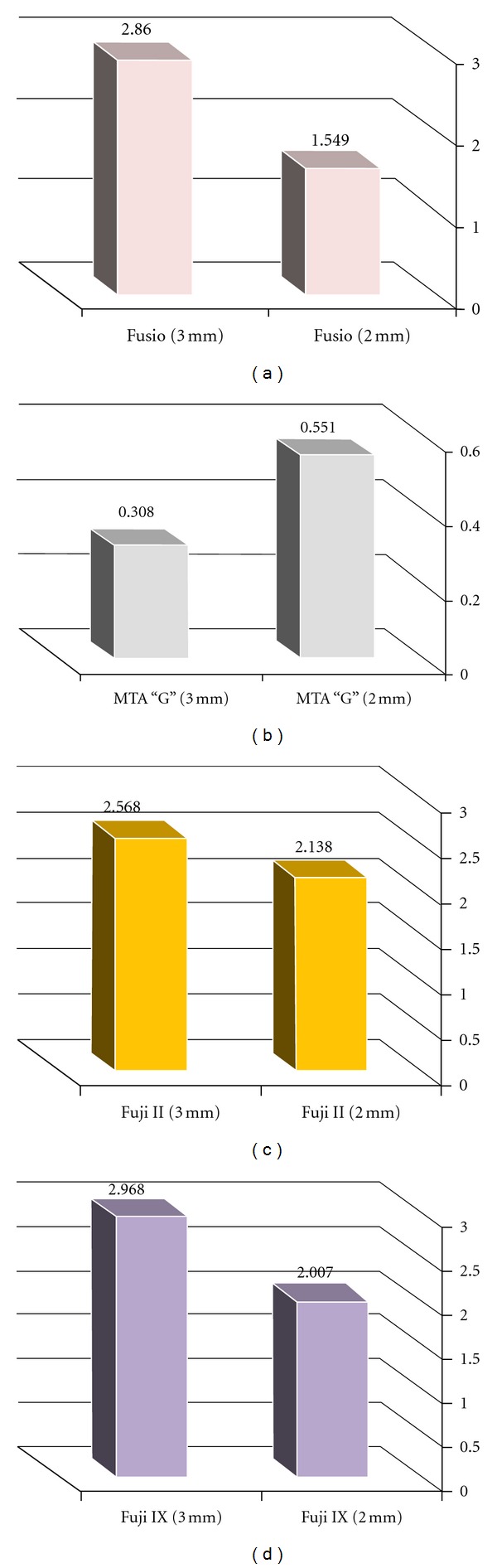
Comparison between linear leakage score in mm for Fusio (a), MTA “G” (b), Fuji II (c), and Fuji IX (d) tested material at 3 mm and 2 mm orifice depths.

Again, a high statistical difference was found in the leakage score between the 2 mm as compared to 3 mm depth in all tested materials (Figures 1(a)–1(d)). The 3 mm depth showed a general trend towards a greater leakage score as compared to the 2 mm depth in all tested materials. The only exception was found in MTA “G” (Figure 1(b)) where the leakage score was higher when the material was placed at 2 mm depth than the 3 mm depth (0.551 mm ± 0.004 mm and 0.308 mm ± 0.08 mm, resp.). This difference was found to be highly significant (*P* < 0.001) (Table 1).

Tables 2 and 3 present the *t*-test for independent samples between each two materials at 2 mm and 3 mm, respectively. These tables gave the values for “*t*” and summarized the results. A high significant difference was found between all materials tested at the two tested depths (*P* < 0.001). On the other hand, a significant difference was found between Fuji II and Fuji IX at 2 mm depth (*P* < 0.01).

## 4. Discussion

Reviewing the literature concerning the depth of the intra-orifice barrier revealed an inconsistency in this issue. Aside from the leakage studies designed specifically to test the effect of orifice cavity depth which were found to be scarce and deficient [24], orifice cavity depths studied varied from a mere indentation [17], 1 mm depth [25], 2 mm depth [18, 25–27], 3 mm depth [16, 22, 28], 3.5 mm depth [19], and 4 mm depth [21]. The present study was designed to investigate the effect of orifice cavity depth on the sealing ability of the four tested materials. This was done through adopting two depths to experiment with, which are 2 and 3 mm. This was based on the recognition that the majority of the previous studies used either of these two depths which seemed more reasonable and suitable for the contemporary barrier materials than the other extremes. Another factor is that we have to consider the possible need for removal of the orifice barrier if retreatment is required. As most of the current barrier materials are based on adhesion, so we can consider that the deeper the intra-orifice barrier material, the more difficult and more risky is its removability. In fact the use of 4 mm depth coronal barrier is too deep as it is not a barrier in the proper meaning of the word and has been mentioned in previous studies only scarcely. In Bailón-Sánchez et al. [21] study, a 4 mm intra-orifice depth was used; this may be because one of their tested materials was cavit.

In discussing their results Parolia et al. [19] stated that they selected 3.5 mm material thickness to seal the canal orifices as it was previously recommended to be the minimum thickness required. However, this was reported in 1978 [29] as the suitable depth of a temporary filling material and not for an intra-orifice barrier. As with the later type, the double seal concept will be completed by a coronal filling material too. 

In the present study methylene blue dye was used as a leakage tracer based on its availability, simplicity of use, as well as its confirmed results. Kubo et al. [30] reported that dyes or radioisotopes are used in 82% of marginal leakage studies. When they investigated the effect of endodontic materials on the optical density of dyes used in marginal leakage studies, they found no significant statistical difference among methylene blue, indian ink, or rhodamine B dye solutions evaluated. In fact methylene blue and rhodamine B dyes both are types of heteropolyaromatic dyes [31].

 In the current study a flowable composite (Fusio), two glass ionomer formulations, namely, Fuji II and Fuji IX, and MTA “G” were tested for their sealing ability in root canals orifices at the prespecified depths.

Irrespective of the orifice cavity depth, generally all tested materials leaked to various degrees. Collectively, the calculated leakage scores for Fuji IX, Fuji II, Fusio and MTA “G” were found to be a mean of: 2.487 mm, 2.353 mm, 2.204 mm, and 0.429 in a descending order. This justifyies the highly statistically significant lowest linear leakage score that was found with MTA “G” at both thicknesses studied, namely, 2 and 3 mm as compared to the rest of materials tested.

Comparable high leakage scores were detected at the 3 mm depth for the other three materials tested, where Fusio liquid composite gave a leakage score between the two Fuji glass ionomers. However, at 2 mm depth a clear trend was recognized where leakage was highest in Fuji II, Fuji IX, and Fusio in a descending order. This means that, for Fusio, linear leakage was affected by the orifice cavity depth where a smaller leakage score was calculated at a 2 mm depth. This difference was found to be statistically significant.

The null hypothesis should then be partially rejected, as in the present study two materials, Fusio and MTA “G”, were significantly affected by orifice cavity depth.

 Fusio is a self-adhesive, flowable composite that was presented with promises on its ability to bond to dentin without a separate adhesive. It was reported from the manufacturer to serve as a dentin replacement. In the present study, the liquid composite used was ranked the third among the high leaky materials in a descending order. Similar results were reported in previous studies [19, 21, 32] irrespective of the difference in leakage testing 11 methodology. A disagreement was however noted in the results of a dye leakage study by Jenkins and Jiang et al. where Esthet flow, beautifil flow, and Filtek Z350 used as orifice barriers did not leak [22, 24].

 The greatest leakage score occurred with conventional glass ionomer Fuji IX “fast” followed by GC Fuji II LC. This result was not speculated. GC Fuji II LC is a light-cured resin reinforced glass ionomer developed for use as a core build up material. As it was reported by the manufacturer, it affects strong chemical bonding to tooth structure. In the present study however, this material resulted in a high leakage score and was ranked the second in respect to maximum leakage among the four tested materials.

Although in a study made by Seiler [33] he found that glass ionomer and resin-modified glass ionomer provided a better coronal seal against *Streptococcus mutans*, this was in comparison to zinc oxide/eugenol coronal restoration. Same result also was found by Delmé et al. [34].

Nonetheless, our results were in harmony with those of Gjorgievska et al. [35]; they reported that both glass-ionomers showed inferior marginal quality and durability with the margins of the resin-modified glass-ionomer slightly superior.

Again, Suresh and Nagarathna [36] evaluated the shear bond strength of Fuji II and Fuji IX before and after saliva contamination. They found that shear bond strengths of both materials were not significantly different from each other when uncontaminated with saliva. On the other hand, salivary contamination resulted in lower bond strengths with respect to Fuji II.

 Results of the present study showed that the MTA “G” thickness (depth of placement) was inversely proportional to the extent of linear leakage. This result was statistically significant. However, our results contradicted that of Parolia et al. [19], with an intra-orifice cavity depth of 3.5 mm, they found that MTA has shown statistically significantly more leakage than LC GIC. In another study, Tetric demonstrated a significantly better seal than Pro Root or Cavit (*P* < 0.0001) irrespective of orifice depth [24].

Our result, on the other hand, was in accordance with that of Rahimi et al. [37], Al-Kahtani et al. [38], and Lawley et al. [39]. Comparing three thicknesses of MTA apical plug, they found that the leakage increased with the decrease in depth. This might be because MTA as a nonadhesive material, behaves differently Tay and Pashley [40] in their paper on 12 monoblocks in root canals elucidated that as a monoblock, MTA does not bond to dentin; however, the good seal of this material is owed to the formation of nonbonding, gap-filling apatite deposits.

## 5. Conclusion

Within the limitations of this study we have tha following.

1. The null hypothesis should be partially rejected, as in the present study two materials, Fusio, and MTA “G”, were affected by orifice cavity depth with regards to their sealing ability.
2. As far as sealing ability is concerned, MTA is the best orifice barrier with the least leakage when placed at 2 or 3 mm depths, and the second material in order is Fusio when placed at 2 mm depth.
3. As the ability to remove the intracanal filling material is one of the ideal requirements for an obturating material, the shorter the orifice barrier depth, the safer its removability when needed.
4. 2 mm orifice cavity depth is a suitable depth for most of the adhesive orifice barrier materials; however, if MTA is going to be used, this might need a 3 mm cavity to affect good sealing ability.

## Figures and Tables

**Table 1:** Descriptive statistics of linear leakage results in mm for the four tested materials at the two specified orifice depths.

Material used	Depth in mm	Mean linear leakage in mm	Standard deviation	Standard error	*t*	*P* value
Fusio	(2 mm)	1.549	0.071	0.05	58.30	<0.001
	(3 mm)	2.86	0.004	0.002		
MTA “G”	(2 mm)	0.551	0.08	0.012	6.92	<0.001
	(3 mm)	0.308	0.077	0.021		
Fuji II	(2 mm)	2.138	0.036	0.025	24.92	<0.001
	(3 mm)	2.568	0.041	0.029		
Fuji IX	(2 mm)	2.007	0.108	0.076	22.11	<0.001
	(3 mm)	2.968	0.085	0.06		

**Table 2:** Results of *t*-test for independent samples between each two materials at 2 mm depth.

Material used (2 mm)	MTA “G”	Fuji II	Fuji IX
Fusio	29.51***	23.40***	11.21***
MTA “G”		57.21***	34.26***
Fuji II			3.64**

*P < 0.05; **P < 0.01; ***P < 0.001.

**Table 3:** Results of *t*-test for independent samples between each two materials at 3 mm depth.

Material used (3 mm)	MTA “G”	Fuji II	Fuji IX
Fusio	104.67***	22.42***	4.01***
MTA “G”		81.92***	73.34***
Fuji II			13.40***

*P < 0.05; **P < 0.01; ***P < 0.001.
